# Transcriptional Profiling Shows Dampening of Interferon Gene Signatures by NAD^+^ Augmentation in Ataxia-Telangiectasia

**DOI:** 10.3390/ijms27135652

**Published:** 2026-06-23

**Authors:** Veronica Suaste, Rebecca Presterud, Anna B. Wennerström, He-Ling Wang, Jianying Zhang, Solveig Osnes Lund, Helle Graneng Holmen, Torben Lüders, Alexander Rowe, Rolf Kristian Berge, Lisa Lirussi, Yohan Lefol, Lene Alsøe, Evandro Fei Fang, Asbjørg Stray-Pedersen, Hilde Loge Nilsen

**Affiliations:** 1 Department of Biosciences, University of Oslo, 0317 Oslo, Norway; veronsua@uio.no; 2 Department of Microbiology, Oslo University Hospital, 0424 Oslo, Norway; 3 Institute of Clinical Medicine, University of Oslo, Norway and CRESCO—Centre for Embryology and Healthy Development, 0316 Oslo, Norway; 4 Institute of Clinical Medicine, University of Oslo, 0318 Oslo, Norway; 5 Department of Clinical Molecular Biology, University of Oslo and Akershus University Hospital, 1478 Lørenskog, Norway; 6 Norwegian National Unit for Newborn Screening, Division of Paediatric and Adolescent Medicine, Oslo University Hospital, 0373 Oslo, Norwayastraype@ous-hf.no (A.S.-P.); 7 Department of Chemistry, University of Bergen, 5020 Bergen, Norway; 8 Norwegian Centre on Healthy Ageing (NO-Age) and Norwegian National Anti-Alzheimer’s Disease (NO-AD) Network, 0316 Oslo, Norway

**Keywords:** Ataxia-Telangiectasia, transcription profiling, NAD^+^, nicotinamide riboside, interferon gene signature

## Abstract

Ataxia-Telangiectasia (A-T) is a multisystem disorder caused by loss of A-T mutated (ATM) protein activity, characterized clinically by immunodeficiency and cerebellar ataxia. ATM is a master regulator of DNA damage responses and loss of ATM function is accompanied by persistent activation of PARP1 leading to depletion of intracellular NAD^+^ and dysfunction of a series of cellular signalling pathways dependent on NAD^+^, providing a mechanistic rationale for NAD^+^ augmentation therapy. We performed a clinical trial of NAD^+^ augmentation with nicotinamide riboside (NR) over 24 months in A-T patients where we observed improved coordination and eye movements in A-T patients. Here, by using peripheral blood mononuclear cells, we performed longitudinal transcriptome profiling to define molecular signatures of A-T and to assess pathway-level responses to NR supplementation. A-T patients exhibited reproducible transcriptomic alterations involving immune, vascular, and inflammatory pathways. NAD^+^ augmentation was associated with suppression of interferon response genes and modulation of networks correlated with neurological improvement. These findings establish systemic molecular signatures of A-T and identify potential blood-based biomarkers that reflect disease processes and therapeutic response, supporting the use of NAD^+^ augmentation as a disease-modifying strategy in A-T by dampening interferon signalling.

## 1. Introduction

Ataxia-Telangiectasia (A-T) is a rare autosomal recessive multisystem disorder caused by biallelic mutations in the Ataxia-Telangiectasia Mutated (*ATM*) gene on chromosome 11q22.3 (OMIM#208900) [1]. Over 1400 unique mutations have been identified. The incidence of A-T is variable by region, with estimates ranging from 1:10,000 in certain parts of Norway [2] to 1:300,000 [1,3]. Heterozygous carriers do not develop A-T but a recent meta-analysis [4] suggested that A-T carriers have a reduced life expectancy due to mortality from cancer and ischaemic heart diseases (RR 1.7, 95% CI 1.2–2.4) [5]. *ATM* encodes a serine/threonine kinase essential for coordination of cellular responses to DNA damage [6,7]. Disease severity of A-T correlates with the function of ATM-kinase with complete loss of ATM kinase activity giving rise to classical A-T disease, characterized by progressive cerebellar ataxia, immunodeficiency, cancer predisposition, and premature ageing [8,9]. Patients with some residual ATM kinase activity generally have milder phenotypes [10].

A central pathogenic mechanism in A-T is the failure to respond to genotoxic stress, including, but not restricted to, single- and double-stranded DNA breaks (SSBs and DSBs, respectively) [9,11,12]. The ATM kinase function is thus essential for B and T lymphocyte development owing to the role of ATM in processing of DSB during class-switch recombination [13] and V(D)J recombination [14,15]. Importantly, as ATM has more than 1000 substrates (including phosphorylation and potentially other types of interactions), it is not surprising that the cellular functions of ATM have extended from genome maintenance to a broad spectrum of cellular functions, such as autophagy, mitochondrial homeostasis, maintenance of cellular redox balance, and neurogenesis [16,17,18,19,20].

The neurological presentation in classical A-T is progressive cerebellar ataxia. The mechanisms underlying the progressive loss of Purkinje neurons and neuromotor symptoms that define A-T are incompletely understood but thought to result from the combined effects of genome instability, oxidative stress, and mitochondrial dysfunction [20,21,22]. In recent years, chronic neuroinflammation involving astrocytes and microglia has emerged as a likely contributor to neurodegeneration in A-T [23,24,25].

We previously demonstrated that persistent activation of an alternative DNA damage sensor poly-(ADP-ribose) polymerase (PARP1) in ATM-deficient cells, *C. elegans* and mouse models, was accompanied by gradual depletion of the oxidized form of nicotinamide adenine dinucleotide (NAD^+^) [22]. NAD^+^ is a critical cofactor for mitochondrial metabolism, DNA repair, and cellular stress responses and, in preclinical models, NAD^+^ depletion resulted in progressive mitochondrial dysfunction, impaired mitophagy, oxidative stress, and neurodegeneration [26]. Consistent with NAD^+^ deletion being a key contributor to these complex phenotypes, we found that supplementation with NAD^+^ precursors, such as nicotinamide riboside (NR), restored mitochondrial function, improved DNA repair capacity, extended lifespan, and attenuated neuromuscular decline in animal models of A-T [22].

NAD^+^ therefore connected both the DNA repair and mitochondrial maintenance phenotypes in ATM-deficient cells and animals, inspiring two pilot studies on NAD^+^ augmentation in A-T. Both studies were open-label, single-armed clinical studies of NR supplementations over four months [27] or 2 years [28]. In both studies, NR increased NAD^+^ levels in peripheral blood and was accompanied by improvements in neuromotor functional scores [27,28], most prominently within the motor coordination and oculomotor control domains [28]. However, inter-individual variability in clinical response remained [28], and the systemic molecular effects of NAD^+^ augmentation in humans with A-T are poorly characterized.

Here, we used transcriptomic profiling to define the molecular landscape of A-T and to identify gene expression signatures associated with NR supplementation. By combining longitudinal sampling with clinical outcome measures, we aimed to identify pathways linked to therapeutic benefit of NAD^+^ augmentation and to establish biomarkers that could inform future clinical trials in A-T.

## 2. Results

### 2.1. Distinct Peripheral Blood Transcriptomic Signature in Patients with Ataxia-Telangiectasia

**Figure 1:**
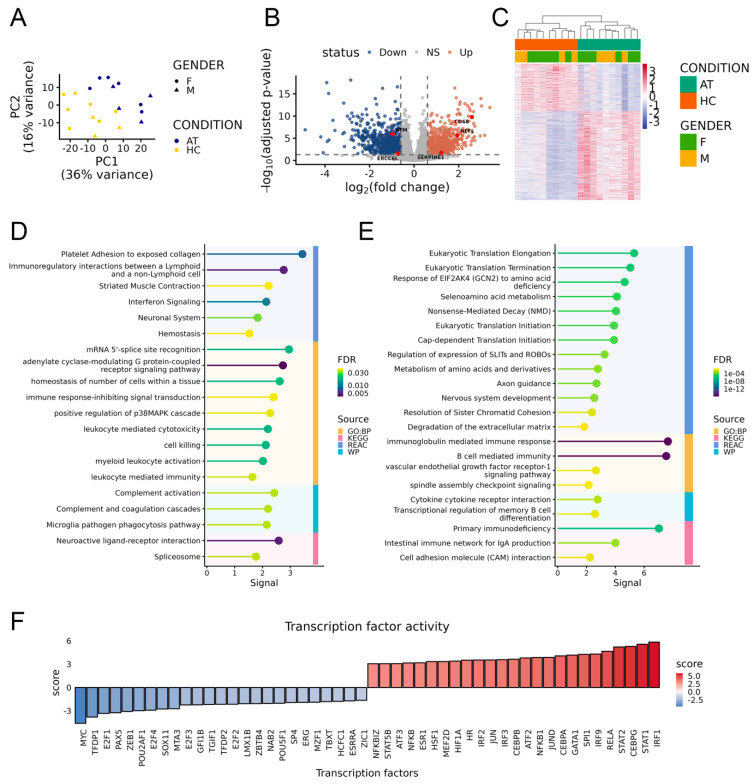
**RNA-seq analysis of A-T samples compared with healthy controls at baseline.** (**A**) Principal component analysis (PCA) based on the top 1000 most variable genes across all samples. (**B**) Volcano plot showing the distribution of fold change (*x*-axis) and log10 (adjusted *p*-value) (*y*-axis) for the comparison of A-T versus healthy controls. Significantly upregulated and downregulated genes in A-T are highlighted (orange, upregulated; blue, downregulated). Dashed lines indicate the thresholds for differential expression: adjusted *p*-value < 0.05, |log_2_FC| > 0.6. Selected genes are labelled. (**C**) Heatmap of differentially expressed genes. Gene expression represented as z-scores, blue to red colour scale. Samples are annotated by condition (A-T/HC) and gender (F/M). (**D**) Selected pathways significantly enriched in over-representation analysis of upregulated genes. (**E**) Selected pathways significantly enriched in over-representation analysis of downregulated genes. (**F**) Transcription factor activity scores for the top 50 regulators. Blue indicates negative activity scores, reduced activity in A-T compared with healthy controls, and red indicates positive scores (increased activity in A-T).

To define systemic molecular alterations associated with A-T, we performed bulk RNA sequencing of peripheral blood mononuclear cells (PBMCs) from ten patients with genetically confirmed A-T and ten age- and gender-matched healthy controls. Principal component analysis (PCA) of the regularized log-transformed counts, considering the 1000 most variable genes across all samples, revealed a clear separation between A-T patients and healthy controls along PC1 and PC2 which explained 36% and 16% of the total variance respectively. The broad dispersion of the samples within each condition indicates substantial variability among individuals, without obvious clustering by gender (Figure 1A).

Differential expression analysis identified 1862 differentially expressed genes (adjusted *p*-value < 0.05, |log_2_FC| > 0.6) between the two conditions out of 14,782 expressed genes. Among these, 1038 genes were upregulated and 824 genes were downregulated in the A-T patients compared with healthy controls (Supplementary Table S1). The volcano plot (Figure 1B) shows the distribution of log_2_ fold change versus statistical significance together with the cut-offs used to define differentially expressed genes. The *ATM* gene is highlighted among the downregulated genes, consistent with A-T, in which many pathogenic variants are loss-of-function mutations and some transcripts are degraded by nonsense-mediated decay of *ATM* transcripts [29]. A few additional genes involved in DNA damage response are also highlighted: upregulation of *FANCA* and *FANCL*, which belong to the Fanconi Anemia complementation group and are involved in DNA cross-link repair and in the maintenance of normal chromosome stability, and downregulation of *ERCC6L*, which plays a role in spindle checkpoint assembly. Interestingly, *PHGDH* encoding, an NAD^+^-dependent enzyme involved in the early steps of L-serine synthesis that connects neurodevelopment, NAD^+^ salvage pathways, and mTOR signalling was among the top downregulated genes.

A heatmap of the 1862 differentially expressed genes, represented as z-scores per gene, is shown in Figure 1C. Hierarchical clustering (Euclidean distance, complete linkage) demonstrates consistent grouping of A-T samples separately from healthy controls, regardless of gender, indicating a robust disease-associated transcriptional signature.

To further investigate transcriptional dysregulation in A-T samples, we inferred transcription factor activity scores by comparing patients with healthy controls. Positive scores indicate higher inferred transcription factor activity in A-T samples relative to healthy controls, whereas negative scores indicate lower activity. The top 25 transcription factors with positive scores and the top 25 with negative scores are shown in Figure 1F. Notably transcription factors IRF1, STAT1/STAT2, NFkB (RELA and NFKB1), and IRF3, key regulators of interferon signalling and innate immune response, showed strong increased activity in A-T patients, whereas scores for B cell transcription factors MYC, E2F1, POU2AF1 and PAXB5 indicate decreased activity in the comparison (Figure 1F).

To identify the biological relevance of the deregulated genes, we performed overrepresentation analysis (ORA). The analysis was conducted separately for upregulated (1038 genes) and downregulated (824 genes) gene sets, using a false discovery rate (FDR) threshold of 0.05 to define statistical significance.

Downregulated genes were significantly enriched in a total of 110 terms across the four queried databases; selected representative terms are shown in Figure 1E, and the complete results are provided in Supplementary Table S2. The biological processes with strongest enrichment signal included “immunoglobulin-mediated immune response” (FDR = 1.1 × 10^−16^), “B cell-mediated immunity” (FDR = 1.2 × 10^−16^) and “Primary immunodeficiency” (FDR = 5.3 × 10^−7^) consistent with the well-established immunodeficiency phenotype in A-T patients [26,30]. We also observed downregulation of pathways related to nervous system development, RNA metabolism, and sister chromatid cohesion. Additionally, translation-related pathways were enriched among downregulated genes, in line with recent reports of translational deregulation in A-T [31].

To identify downregulated processes more directly linked to ATM loss, we further filtered enriched pathways to those explicitly containing ATM. These pathways predominantly involved proliferation and differentiation of immune system cells, as well as chromosome stability, including “negative regulation of chromosome separation and segregation” and “sister chromatid segregation and separation.” A complete list of these ATM-containing pathways and their statistics is provided in Supplementary Table S4.

As for upregulated genes, these were significantly enriched in only 22 terms across the four queried databases; selected representative terms are shown in Figure 1D (complete results are provided in Supplementary Table S3). The most strongly enriched terms included “Interferon Signalling” (FDR = 0.01), which has been widely reported in A-T studies [21,22], as well as immune processes such as “immune response-inhibiting signal transduction” (FDR = 0.04) and “complement activation” (FDR = 0.03). We also observed enrichment of pathways related to neuronal dysregulation including “Neuronal System” (FDR = 0.02) and “Neuroactive ligand-receptor interaction” (FDR = 0.004) consistent with the neurological involvement in A-T.

Interestingly, the *SERPINE1* gene, which has been linked to the severity of A-T [30], was present in upregulated pathways such as “Hemostasis” (FDR = 0.04) and “Complement and coagulation cascades” (FDR = 0.035). Additional significant terms related to cell killing and microglial phagocytosis were also identified, driven by shared genes *FCGR1A*, *TREM1*, and *PIK3R6*.

Taken together, these results suggest activation of interferon and complement/coagulation pathways alongside altered neuronal signalling, potentially linking immune dysregulation to neurodegenerative processes in A-T.

### 2.2. Redox and Mitochondrial Function Markers in Response to NR Supplementation

ATM is involved in mitochondrial maintenance and acts as a sensor of mitochondrial ROS [18]. Genes involved in complex I assembly, oxidative phosphorylation and the mitochondrial electron transport chain (*ATP8*, *ATP9*, *COX2*, *GLDC*, *MT-ND3*, *MT-CO2 MT-CO3*, *CSKMT*) were enriched among the upregulated genes (Figure 1E).

**Figure 2:**
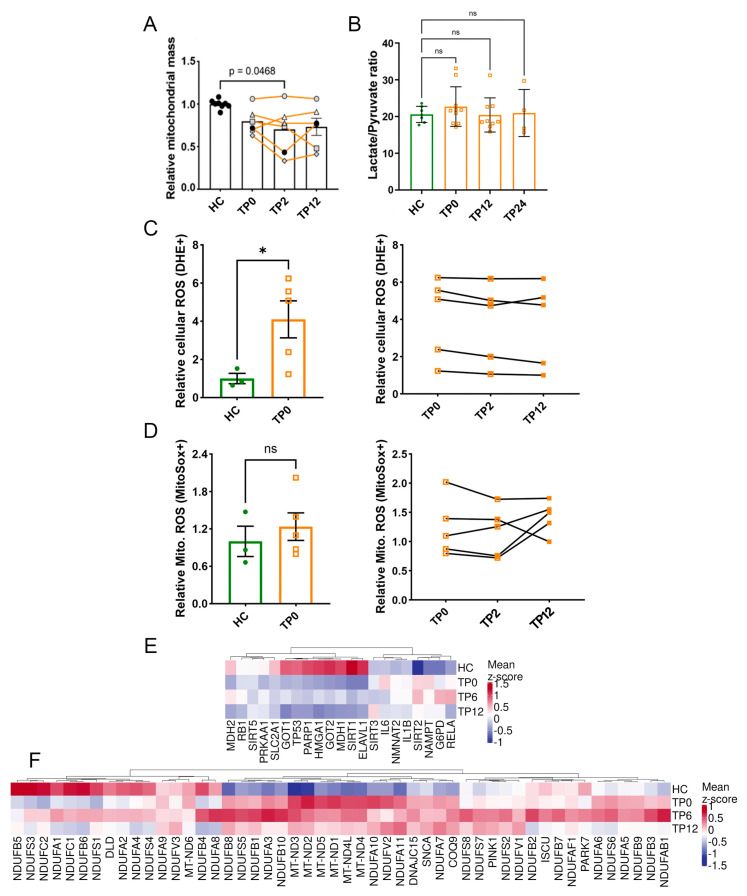
**NAD^+^ augmentation does not alter redox and mitochondrial function markers.** (**A**) Relative mitochondrial mass measured in PBMC from healthy controls (HC) and A-T patients at baseline (TP0) and after NR supplementation for 2 and 12 months. Data are presented as mean ± SD. *p* = 0.0468. (**B**) Lactate/pyruvate ratio in healthy controls (**C**) and A-T patients at baseline (TP0) and after NR supplementation for 12 and 24 months. ns = not significant. (**C**) Relative cellular ROS levels (DHE) in healthy controls (HC) and A-T patients at baseline (TP0) (left panel, * *p* < 0.05). Changes in cellular ROS levels in A-T patients after 2 and 12 months of NR supplementation, right panel. (**D**) Relative mitochondrial ROS levels (MitoSOX) in healthy controls (HC) and A-T patients at baseline (TP0) (left panel, ns). Changes in mitochondrial ROS levels in A-T patients after 2 (TP2) and 12 months (TP12) of NR supplementation, right panel. (**E**,**F**) Heatmaps showing average z-score gene profile over time on a blue–red colour scale for NAD signature genes in A-T patients and healthy controls; (**E**) GO:BP mitochondrial electron transport NADH to ubiquinone; (**F**) WikiPathways “NAD metabolism in oncogene-induced senescence and mitochondrial dysfunction-associated senescence”.

To better interpret functional implications of these gene expression changes, we used flow cytometry to assess whether mitochondrial dysfunction was also observed in PBMC from A-T patients as in ATM-defective neurons and animal models (Supplementary Figure S1). A-T patients at baseline had reduced mitochondrial mass compared to healthy controls (Figure 2A). Lactate/pyruvate ratio (Figure 2B) and several other mitochondrial metabolites in plasma (Supplementary Figure S2) were indistinguishable from controls. Cellular ROS levels were significantly elevated in the patients compared to controls (Figure 2C, left panel), which is consistent with ATM functioning as a redox regulator. Mitochondrial ROS levels were not elevated in the patients at baseline (Figure 2D, left panel).

Interestingly, NR supplementation did not affect cellular ROS levels in PBMC. Repeated-measures one-way ANOVA (Figure 2C, right panel) revealed no significant differences among groups (F(1.282, 5.12) = 3.657, *p* = 0.1099, Greenhouse–Geisser corrected) as significant inter-individual variability was observed (F(4,8) = 303.5, *p* < 0.0001). Paired *t*-test showed no significant difference in cellular ROS between baseline (0 months, 0M) and 12 months (12M) (mean difference = −0.34, 95% CI −0.84 to 0.15, t(4) = 1.92, *p* = 0.128, η^2^ = 0.48) (Supplementary Figure S1C). For mitochondrial ROS levels (Figure 2D, right panel), repeated-measures one-way ANOVA revealed no significant change in mitochondrial ROS across the three time points (F(1.183, 4.734) = 1.11, *p* = 0.358, η^2^ = 0.217). Large inter-individual variability was observed (F(4,8) = 4.59, *p* = 0.032) and a paired *t*-test showed that mitochondrial ROS levels were not significantly different between baseline (0M) and (12M) (t(4) = 0.85, *p* = 0.443) (Supplementary Figure S1D).

Moreover, whereas mitochondrial function was enriched among the gene sets differing between A-T patients and controls, they were not prominently represented among the gene sets that were consistently changed upon NR supplementation. This is illustrated in heat maps of hallmark genes. Interestingly, the hallmark gene sets “NAD metabolism in oncogene induced senescence” (Figure 2E) and “mitochondrial dysfunction-associated senescence” (Figure 2F), while being suppressed in A-T patients compared to control, were not affected by NR supplementation. Thus, biomarkers reflecting a mitochondrial dysfunction phenotype were not obviously affected by NR in PBMCs from A-T patients.

### 2.3. Transcriptomic Trajectories with NR Supplementation

**Figure 3:**
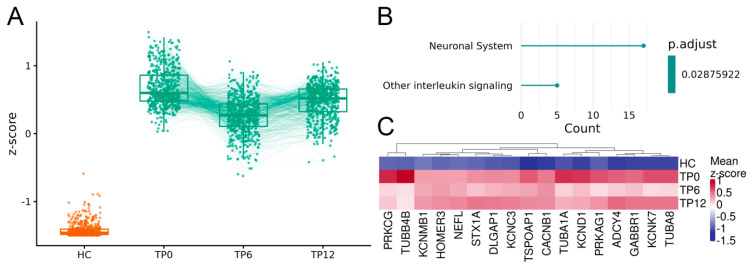
**Longitudinal gene expression dynamics in A-T patients on NR supplementation.** (**A**) Trajectory of cluster 2 genes across three timepoints in A-T patients, with healthy controls (HC) included on the *x*-axis for comparison. The *y*-axis shows the mean gene expression per group, represented as z-score averages. Each dot represents an individual gene within cluster 2. (**B**) Top pathways significantly enriched in cluster 2, identified using the WikiPathways (WP) database. (**C**) Heatmap of genes at the intersection between the “Neuronal System” pathway and cluster 2, showing z-score gene expression over time on a blue–red colour scale. Healthy controls (HC) are included for reference.

To identify genes, and particularly biological pathways, showing evidence of temporal variation in A-T patients on NR supplementation, we applied a likelihood ratio test (LRT) across timepoints and healthy controls, followed by divisive hierarchical clustering approach (Supplementary Tables S6 and S7). This analysis yielded two main clusters (Supplementary Figure S3A); cluster 2 exhibited the most pronounced changes over time, comprising 551 genes (Figure 3A). We then performed over-representation analysis on cluster 2 and identified significant enrichment for pathways related to the neuronal system and interleukin signalling (Figure 3B). Given the relevance of the neuronal pathology in A-T, we specifically examined the genes contributing to neuronal system enrichment. The 17 genes at this intersection include neurofilament light chain (*NEFL*), which encodes NfL and is considered a biomarker of neurodegeneration (Figure 3C). Their temporal expression trajectories, represented as z-scored expression values, tended to move closer to that of healthy controls at the 6-month time point, but remained at elevated levels (Figure 3C).

**Figure 4:**
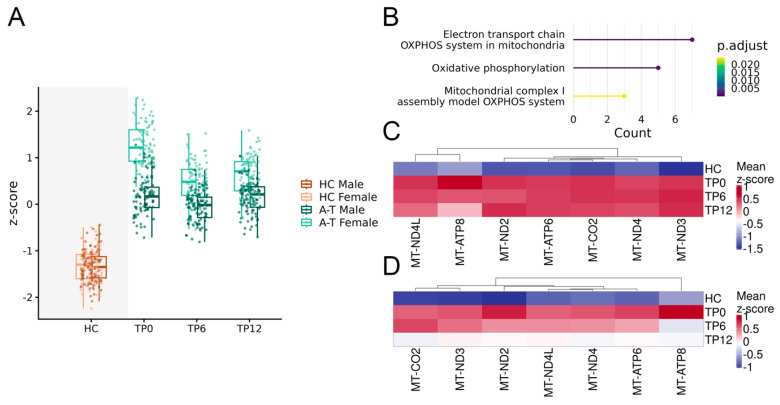
**Longitudinal gene expression dynamics in male and female A-T patients on NR supplementation.** (**A**) Trajectory of cluster 5 genes across three timepoints in A-T patients, stratified by gender, with healthy controls (HC) included on the *x*-axis for comparison. The *y*-axis shows the mean gene expression per group (timepoint and gender), represented as z-score averages. Each dot represents an individual gene within cluster 5. (**B**) Top pathways significantly enriched in cluster 5, identified using the Reactome (REAC) database. (**C**) Heatmap of genes at the intersection between cluster 5 and the three pathways in (**B**) for male A-T patients across time. (**D**) Heatmap of genes at the intersection between cluster 5 and the three pathways in (**B**) for female A-T patients across time.

To assess whether differences related to gender could refine the characterization of longitudinal transcriptomic responses to NR supplementation, we repeated the clustering analysis grouping the samples by gender (Supplementary Table S8). This approach identified nine gene clusters (Supplementary Figure S3B), among which the female subgroup showed a trend of expression profiles toward those of healthy controls after NR supplementation, particularly in cluster 5 (Figure 4A). Pathway over-representation analysis of cluster 5 genes indicated significant enrichment of mitochondrial processes, including mitochondrial electron transport chain, oxidative phosphorylation, and mitochondrial complex I assembly (Figure 4B). Cluster 5 displayed the strongest temporal shift, with a marked change between baseline (TP0) and 6 months (TP6). Heatmap representations of the seven genes contributing to these mitochondrial pathway enrichments show movement of gene expression in the direction of healthy controls but most evidently in female patients (Figure 4C,D).

### 2.4. Interferon and Inflammatory Gene Signatures Correlate with Clinical Improvement

We previously showed that clinical benefit of NR supplementation could be seen in neuromotor test scores, such as A-T Neurological Examination Toolkit (A-T NEST) and Scale for Assessment and Rating of Ataxia (SARA) total scores [28]. However, while improvement of motoric scores was seen in most A-T patients included in our study, there were inter-individual differences both in total scores and with respect to individual test items where response could be measured [28]. To identify molecular pathways associated with clinical response in an unbiased manner, we performed weighted gene co-expression network analysis (WGCNA) integrating transcriptomes of individual patients and their biomarker scores (NfL) or neuromotor test scores as determined earlier [28].

**Figure 5:**
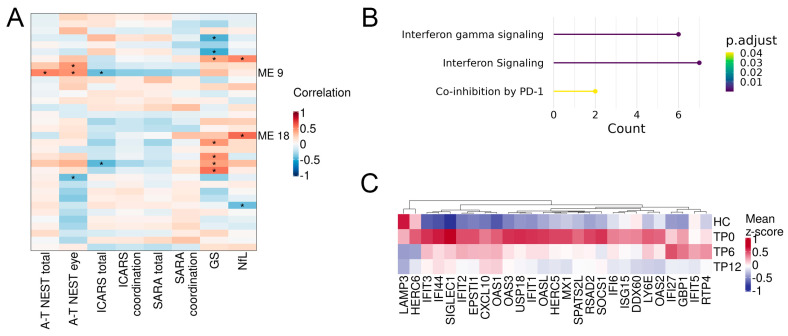
**Weighted gene co-expression network analysis (WGCNA) in A-T patients after NR supplementation.** (**A**) Module–trait correlation heatmap. It displays correlation between modules and neuromotor test scores (A-T NEST, ICARS, SARA and GS) or NfL from A-T patients. An asterisk (*) in the cell indicates that the correlation is statistically significant (*p*-value < 0.05). Module annotated (ME9) correlates significantly with three test scores. (**B**) Top pathways significantly enriched for module 9, identified using the Reactome (REAC) database. (**C**) Heatmap displaying gene expression of the curated interferon-stimulated genes (IGS) across healthy controls (HC) and A-T patients at base line (0M) and after NR supplementation for 6 (TP6) and 12 (TP12) months.

WGCNA identified 34 distinct co-expression modules. We correlated module eigengenes (MEs) with neuromotor test scores and NfL measurement from A-T patients across time. A heatmap summarizing these correlations and their statistical significance is shown in Figure 5A. The module eigengene 9 (ME9) showed the strongest positive correlation with three clinical scores including A-T NEST total (r = 0.5, *p* = 0.01), while ME18 yielded the strongest positive correlation with NfL (r = 0.57, *p* = 0.001). SARA and ICARS scores were not significantly correlated with any modules. Nevertheless, the opposite directionality of their correlations with ME9 was consistent with the clinical interpretation of these scales, as clinical improvement is reflected by higher scores in A-T NEST but by lower values in ICARS (International Cooperative Ataxia Rating Scale) and SARA. In this sense, higher ME9 expression is associated with lower neurological impairment across all three scales.

We further investigated module 9, by performing overrepresentation analysis on the genes belonging to this module (30 genes). The results revealed significant enrichment of the pathways “Interferon Signalling” (adjusted *p*-value = 4.4 × 10^−5^) and “Interferon gamma signalling” (adjusted *p*-value = 3.5 × 10^−6^) driven mainly by GBP family genes (*GBP1*, *GBP2*, *GBP3*, *GBP4*, *GBP7*), *STAT1* and *MAP2K6* (Figure 5B). To corroborate the significance of these genes within the module, we computed intramodular analysis metrics: module membership (MM) as the correlation of the module eigengene and the gene expression profile and gene significance (GSig) as the absolute value of the correlation between the clinical score and the gene expression profile. When filtering top gene (MM > 0.8 and GSig > 0.5) the same GBP family genes, *STAT1* and *MAP2K6* remain significant together with other relevant genes, such as APOL gene family genes (*APOL3*, *APOL4*, *APOL6*) involved in innate immunity and programmed cell death, *SLAMF8* involved in the regulation of inflammatory responses, *CASP7* involved in managing cell death and membrane repair, and *CD274* (*PD-L1*) and *IDO1* which are crucial for immune tolerance—complete list and metrics are provided in Supplementary Table S5.

To further analyze NR supplementation in relation to the interferon signalling pathway, which was enriched in the A-T patients (Figure 1D) and correlated with neurological impairment as per the A-T NEST score, we took a curated set of interferon-stimulated genes (ISGs) and examined their expression profiles. Expression values, represented as average per-gene z-scores, were compared between healthy controls and A-T patients at baseline and under NR supplementation (Figure 5C). The heatmap reveals an elevated interferon gene signature in A-T patients at baseline compared to healthy controls and a partial suppression of this signature after 6 and 12 months on NR supplementation.

In a similar manner, we investigated module 18 (50 genes), which presented the strongest correlation with NfL, a biomarker for neurodegeneration (Figure 5A). Notably, the *NEFL* gene, encoding NfL, was also identified as upregulated in A-T patients (log_2_FC = 1.93, *p*_adj_ = 1.9 × 10^−6^). Overrepresentation analysis of module 18 revealed enrichment of “Axon development” (*p*_adj_-value = 0.03) and “negative regulation of cytokine production” (*p*_adj_ = 0.04) pathways—Supplementary Figure S4. These enrichments were driven mainly by *TNFRSF21* and *PTPRS*, which are genes shared by both pathways, as well as by *VASH2*, *EPHB1*, *COBL* and *GPM6B*, in the case of “axon development”. *TNFRSF21* promotes apoptosis, negatively regulates the production of immunoglobulins in response to antigens, and mediates neuronal degeneration and axon pruning [32]. PTPRS plays a key role in neural development, synapse formation, and axon guidance [33]. The positive correlation of module 12 with the NfL levels indicates that higher expression of the genes is associated with higher NfL concentration, consistent with increased neuroaxonal damage. Together, these findings suggest that module 18 captures transcriptional patterns related to axonal integrity and neuroinflammation associated with the neurodegenerative burden in A-T. With respect to NR supplementation [28], we reported that there was no change in NfL levels in the participants during the treatment [28].

In conclusion, we identified treatment-sensitive gene sets supporting the broader concept that NAD^+^ augmentation can partially suppress interferon gene signatures in A-T which correlates with improvement in neuromotor scores.

## 3. Discussion

Ataxia-Telangiectasia is a complex multi-organ disorder with pathophysiology extending beyond defective DNA damage response to encompass chronic inflammation, mitochondrial dysfunction, and systemic metabolic dysregulation. In this study, we used longitudinal transcriptomics data to define molecular signatures of A-T in peripheral blood and to examine how these pathways are modulated by NAD^+^ augmentation with NR.

We show that A-T is associated with reproducible alterations in gene expression patterns. At baseline, A-T patients exhibited a robust and reproducible peripheral blood transcriptomic signature distinctive from healthy controls. Diagnosis was the factor contributing most to the variations observed and female and male patients clustered together.

Downregulated genes were dominated by genes related to B cell development, which is consistent with the well-established immunodeficiency phenotype of classical A-T. However, we observed upregulation of innate immune and inflammatory pathways, interferon signalling, and p38 MAPK signalling. The dominance of interferon signalling was apparent from transcription factor activity analyses returning interferon regulatory factors 1, 3, and 9 (IRF1/3/9) at top upstream regulators. IRF3 induces expression of type I interferons and is the central transcription factor activated following STING activation [34,35]. ISGs are also activated through the JAK-STAT pathway via the ISGF3 complex, composed of STAT1, STAT2, and IRF9, which all have high transcription factor activity in A-T patients at baseline. Other transcription factors such as NFkB (RELA and NFKB1), HIF1A, ATF3, and ATF2 (Figure 1F) have been associated with several cellular stress states, oxidative and inflammatory stress, and are also consistent with high cellular ROS and mitochondrial dysfunction in A-T [36].

Our data showing higher expression of a curated set of ISGs is consistent with findings from other groups reporting elevation of type I and II interferon signatures in peripheral blood of A-T patients [30,37]. Relevance of the IGS signatures is supported by elevated levels of SERPINE1/PAI-1 and other inflammatory mediators in plasma of patients with A-T [30,37,38].

Bulk transcriptome analyses did not allow us to predict whether the enriched interferon response was related to type I or II interferons. A study by Reichenbach and colleagues [39] examined the IL-12/interferon-γ (IFN-γ) axis specifically in monocytes and INF-γ-producing cells (T and NK cells) after challenging cells from whole blood from A-T patients with various types of bacterial products: LPS, heat-killed Staphylococcus aureus and whole live BCG and IL-12. They found that the INF-γ production by T and NK cells was impaired compared to controls, whereas the monocyte function seemed not to be affected. The biomaterial used in the study by Reichenbach and colleagues are comparable to the samples used in our study and the results may at first appear conflicting, but our study did not include any type of stimulation of immune cells. It is also worth noting that the transcription factor IRF1, which had a high activity score in the A-T patients at baseline, functions upstream of interferons, inducing type I and II interferon expression, but also downstream of interferons, being induced by interferons (type I and II). One can therefore speculate that both type I and type II interferons are produced in PBMCs from A-T patients.

It is possible that interferon stimulation is a direct cause of the DNA repair defect as A-T cells have increased levels of DNA damage and genome instability, giving rise to cytosolic damaged self-DNA, which is sensed and acted upon by the cGAS-STING pathway [40,41,42]. This leads to production of type I interferons via IRF3/IRF7. Type I interferons are secreted and autocrine binding to their cell membrane-bound receptors (IFNAR1/IFNAR2) initiates the assembly of the ISGF3 complex (IRF, STAT1, STAT2), which induces expression of ISGs [42]. In general, type I interferons are produced by several of the immune cells; whereas, type II interferons are primarily expressed by NK, NKT and T cells [43]. Induction of type I interferon is primarily induced by cytokines (e.g., IL-12), pattern recognition receptors (PRRs) and antigen stimulation via the T cell receptor [43]. From research on cancer cells, it is known that chronic type I interferon signalling drives immune exhaustion, especially caused by exhaustion of T cells [42]. Since T cells, together with NK and NKT cells, are the primary source of type II interferon (INF-γ), this may explain why Reichenback and colleagues found that the INF-γ production was impaired in IFN-γ-producing cells in A-T patients. We cannot from our analysis assess the origin of expression of activation markers for type I interferon and IFN-γ responses, CD169 (SIGLEC-1) and CD274 (PD-L1), as several of the cell types within PBMCs may express these genes. CD169 is primarily expressed by the monocytes and macrophages and thus most likely originates from monocytes in our data.

In our study, multiple lines of evidence converge on the role of interferon signalling also as a dominant process affected by NR supplementation. Differential expression and overrepresentation analyses revealed robust enrichment of pathways related to interferon among upregulated genes, accompanied by increased inferred activity of transcription factors associated with interferon signalling, such as IRF1, IRF3 and STAT1. A curated set of ISGs showing a pronounced baseline elevation in A-T patients, and a co-expression module (ME9) enriched for interferon pathways, and correlated with neurological scores. At first glance, the positive association between ME9 expression and better neurological status appeared counterintuitive, given the detrimental effects often attributed to chronic interferon activation. However, closer inspection of ME9 indicated that it includes not only classical ISGs (*STAT1*, *GBP1/2/3/4/7*, *APOL2/3/4/6*) but also several genes implicated in negative regulation of interferon signalling and immune tolerance, such as *IDO1*, *PLD1*, *TRIM69* and *CASP7* (Supplementary Table S1). This together raises the possibility that ME9 reflects a compensatory response to persistent interferon drive, in which upregulation of tolerance pathways helps restrain excessive inflammation and, therefore, a more effective regulatory state, consistent with its association with lower neurological impairment. Expression of mRNA for *CD169* and *CD274* was reduced after NR supplementation but still elevated compared to healthy controls (Figure 5C and Supplementary Table S1). Interestingly, some gene sets also correlated with GS scores, where we found no significant improvement of motoric scores [28].

Interferon stimulation may also be activated because of mitochondrial dysfunction. In vitro experiments have demonstrated that depletion of mitochondrial NAD^+^ leads to release of mitochondrial DNA to the cytosol which exacerbates the activation of the cGAS-STING pathway and thus the production of type I interferons [44,45]. However, a gene expression signature consisting of transcription factor ATF3 regulated genes was also proposed as a biomarker in Cockayne syndrome, suggesting that DNA repair deficiency activates inflammatory pathways also independent of cGAS-STING [46]. These findings align with recent reports suggesting that ATM deficiency leads to activation of ISGs through collaborative action of the cGAS-STING, p38MAPK, and p53 pathways in response to persistent DNA damage in ATM-deficient lung fibroblasts [47]. Importantly, we demonstrate in A-T patients in vivo that NR supplementation dampens interferon signalling as the gene expression of ISGs were downregulated after NR supplementation. The expression of most of the ISGs was reduced after 6 months of NR supplementation and further reduced after 12 months but expression levels for most of the genes were still at somewhat higher levels than in HC. Taken together, this suggests that NR dampens the chronic interferon signalling and response in A-T patients. Similarly, Lynch et al. [48] showed that suppression of an interferon gene signature score calculated based on expression of six genes, *IFI27*, *IFI44L*, *ISG15*, *IFIT1*, *RSAD2*, and *SIGLEC1* measured by qRT-PCR, was dampened by triheptanoin which corrects metabolic stress in patients with A-T. Also, in the triheptanoin intervention study, suppression of ISGs was associated with improvement in neuromotor functional scores.

Taken together, the combination of (i) a strong interferon signature at baseline, (ii) an interferon-related module enriched for regulatory genes that tracks with better neurological scores, and (iii) attenuation of ISG expression under NR supplementation, supports a model in which fine tuning, rather than complete suppression of interferon signalling, may be beneficial in A-T. Because NR can increase cellular NAD^+^ levels, a metabolite with recognized roles in both cancer metabolism and immune regulation, concerns about potential pro-tumorigenic or tolerance-promoting effects are legitimate. Our study was too small and too short (two years) to evaluate malignancy risk or comprehensive immune phenotyping, but we did not observe any obvious signal from the transcriptomes consistent with these phenomena. Therefore, long-term surveillance and targeted mechanistic studies will be necessary to fully clarify the therapeutic relevance and safety of targeting interferon pathways with NAD^+^ boosting strategies in A-T.

Beyond ISGs, our analyses identified consistent alterations in genes related to vascular development, calcium handling, and neuronal connectivity consistent with earlier studies [49]. Several downregulated transcripts encode proteins involved in axon guidance, synaptic organization, and neurodevelopment. Although these measurements were obtained from peripheral blood cells, similar gene sets have been implicated in cerebellar development and neurodegenerative processes [35,47,49], suggesting that systemic transcriptional changes may reflect central nervous system pathology [50].

The effects of NR on redox balance and mitochondrial stress responses were less obvious compared to changes in type I interferon and innate immune gene signatures. Cellular ROS levels were elevated in the A-T patients at baseline compared to control, which is consistent with ATM functioning as a redox regulator. However, comparing patients at baseline and 6 or 12 months after NR supplementation, no significant differences were found in cellular ROS or mitochondrial ROS. Yet, the effect size estimations (Supplementary Figure S1C,D) suggest that there might be a biological effect that may require a larger sample size to confirm. It is also likely that NAD^+^ augmentation has greater effects on mitochondrial function in neurons [22,36] than in PBMC. On the transcriptome level, genes and pathways related to mitochondrial function were largely found among the upregulated genes at baseline. Functional measurement of mitochondrial dysfunction in the patient PBMCs at baseline showed reduced mitochondrial mass yet mitochondrial ROS, lactate/pyruvate ratio and the levels of several other mitochondrial metabolites were indistinguishable from healthy controls. Taken together, this might suggest that the upregulation of transcripts related to mitochondrial function seen in peripheral blood might be a compensatory upregulation to mitochondrial dysfunction.

A key objective of this study was to identify molecular signatures associated with clinical outcomes. WGCNA identified gene modules that correlated with neurological scores and plasma neurofilament light (NfL) levels. These findings suggest that peripheral blood transcriptomic profiles may capture biologically relevant processes linked to neurological function in A-T. The association between inflammatory gene networks and NfL further supports a connection between systemic immune activation and neuroaxonal injury.

Immune cells actively interact with the central nervous system, infiltrate the brain, and contribute to neurodevelopment, homeostasis, and disease processes [51,52,53]. PBMCs represent attractive biomaterial for studying neurodegenerative diseases because they can be obtained with relatively simple and minimally invasive procedures, which enable repeated sampling over time [54,55,56]. In A-T specifically, transcriptomic profiling of PBMCs has already been used to define subgroups of A-T beyond mild and classic phenotypes, to identify novel peripheral protein features that may represent biomarker candidates for disease classification, and to monitor therapy response [49]. There are, however, clear limitations as PBMCs consist of heterogeneous mixtures of cell types, and their transcriptional profiles provide only an indirect view of cerebellar pathology. As such, PBMC-derived transcriptomes may reflect systemic immune dysfunction without fully capturing the neurodegenerative process. However, given the importance of interferon signalling in A-T, PBMCs show promise as a minimally invasive and biologically relevant method to track inflammatory activity and treatment response in A-T.

Although exploratory, these results highlight the potential of blood-based multi-omics profiling to identify biomarkers of disease activity and therapeutic response in A-T, but findings will require validation against clinical endpoints and neuroimaging or cerebrospinal fluid studies to confirm their relevance to the neurological disease course.

A central premise of NR supplementation in A-T is that chronic PARP1 activation depletes intracellular NAD^+^, thereby impairing mitochondrial function, redox balance, and DNA repair capacity in neurons in preclinical models [22]. It is not expected that peripheral blood fully reflects molecular processes occurring in the central nervous system. In fact, CD38 and not PARP1 is the main consumer of NAD^+^ in blood cells [57]. Still, the transcriptome of A-T patients encompasses footprints of all the known disease mechanisms. The correlation of neurological outcomes with suppression of interferon gene signature suggests that NR might modulate disease-relevant mechanisms. Future studies should ideally integrate measurements in peripheral blood with cerebrospinal fluid biomarkers and neuroimaging to further elucidate mechanisms of disease modification.

## 4. Materials and Methods

### 4.1. Study Design and Participants

This study was conducted as a single-arm, open-label observational intervention trial with longitudinal clinical and molecular follow-up [28]. The study was approved by the Regional Committee of Ethics in Norway (REK 2019/417) and the data protection office at both Akershus University Hospital and Oslo University Hospital and registered at Clinical Trials.gov (NCT04870866). All study participants [28] had molecular-confirmed A-T diagnosis. Of a total of 13 patients enrolled in the study, twelve had classical A-T phenotype, one was classified as variant A-T. Of 13 enrolled, two participants were excluded due to secondary conditions unrelated to the intervention, but their symptoms or concurrent diseases were not worsened by NR supplementation; additionally, one individual chose to withdraw after 12 months due to loose stools. The present study is based on data from the 10 patients (Supplementary Table S9) that completed the NR supplementation study where longitudinal neuromotor test scores and biomarker measurement are available from baseline, and 6 and 12 months of NR supplementation [28]. All had received standardized doses (2 × 250 mg/day) of nicotinamide riboside.

### 4.2. PBMC Isolation and Preparation

Peripheral venous blood was collected into Vacutainer™ CPT™ tubes (BD Biosciences, Franklin Lakes, NJ, USA) containing sodium citrate. Peripheral blood mononuclear cells (PBMCs) were isolated according to the manufacturer’s protocol, cryopreserved in fetal bovine serum supplemented with 10% DMSO, and stored in liquid nitrogen until analysis. Metabolites in CPT-plasma were measured as previously reported [58].

### 4.3. Flow Cytometric Analyses of Cellular and Mitochondrial ROS

Cryopreserved PBMCs were rapidly thawed, washed, and allowed to recover for 2 h at 37 °C in RPMI 1640 supplemented with 10% fetal bovine serum. Cellular ROS was measured using dihydroethidium (DHE; 5 µM, 30 min at 37 °C). Mitochondrial ROS was measured using MitoSOX Red (5 µM, 10 min at 37 °C) (M36008, Invitrogen, Thermo Fisher Scientific, Waltham, MA, USA). Cells were washed twice with PBS and analyzed immediately by flow cytometry using a BD FACSCanto™ instrument (BD Biosciences, San Jose, CA, USA). Data were analyzed with FlowJo v10.8.1. Thawed PBMCs were washed with PBS and incubated with 5 μM DHE in serum-free RPMI 1640 for 30 min at 37 °C in the dark. After incubation, cells were washed twice with PBS and immediately analyzed by flow cytometry. DHE fluorescence was detected using 518 nm excitation and 606 nm emission wavelengths. PBMCs were incubated with 5 μM MitoSOX Red in serum-free RPMI 1640 for 10 min at 37 °C in the dark. Following incubation, cells were washed twice with warm PBS and analyzed immediately by flow cytometry. MitoSOX Red fluorescence was detected using 396 nm excitation and 610 nm emission wavelengths. The gating strategy was as follows: initial gating on forward scatter (FSC) vs. side scatter (SSC) to exclude debris and select the main PBMC population; doublet discrimination using FSC-Height vs. FSC-Area to ensure single-cell analysis; for cellular ROS analysis, DHE-positive cells were identified on a histogram of DHE fluorescence intensity; for mitochondrial ROS analysis, MitoSOX™ Red-positive cells were identified on a histogram of MitoSOX™ Red fluorescence intensity.

Results were expressed as the relative frequency of positive cells within the population. Data are presented as mean ± standard error of the mean (SEM). Statistical significance was determined using both paired and unpaired Student’s *t*-tests. Repeated-measures one-way ANOVA tests were used to compare NR treatment duration effects within groups, while unpaired *t*-tests were used for comparisons between A-T patients and healthy controls. *p* values < 0.05 were considered statistically significant. All statistical analyses were performed using GraphPad Prism software (version 11.0.0 (93)).

### 4.4. RNA Sequencing

Total RNA was extracted from PBMCs using the RNeasy Micro Kit (QIAGEN, Hilden, Germany). RNA quality was assessed using a TapeStation instrument (Agilent Technologies, Santa Clara, CA, USA), and samples with RNA integrity number (RIN) > 7 were used for library preparation. RNA libraries were prepared using the Illumina Stranded Total RNA Prep with Ribo-Zero Plus kit (Illumina, San Diego, CA, USA) and sequenced on an Illumina platform to generate paired-end reads (2 × 100 bp). Raw sequencing data have been deposited in the Zenodo public repository (accessible through https://doi.org/10.5281/zenodo.20662078).

### 4.5. RNA Sequencing Data Processing

As a first step, quality control of the raw sequencing reads was performed using FastQC (v) [59]. Based on this assessment, we detected adapter contamination and used cutadapt (v) [60] to remove adapter sequences. A final quality check was then performed with FastQC. Pre-processed reads were aligned with the human genome reference GRCh38, Ensembl release 109 [61] using STAR aligner [62], with gene annotation from the same Ensembl release. Quantification of counts at the gene level was performed during the alignment step using --quantMode GeneCounts parameter. For downstream analysis, we used the counts reported in the fourth column of the STAR gene quantification output, corresponding to a reverse-stranded library orientation.

### 4.6. Differential Gene Expression Analysis

For this analysis, we used counts from the ten A-T patients at baseline and ten healthy controls that matched in terms of gender and age (Supplementary Table S9). Differential gene expression analysis was performed using DESeq2 [63]. We included gender as a categorical covariate in the design formula to account for potential variability related to gender. Genes with low counts (<15) for at least 50% of all samples (healthy and A-T patients) were filtered out prior to analysis. From the results, genes were considered differentially expressed if they satisfied both of the following two conditions: |log_2_ (fold change)| > 0.6 and adjusted *p*-value < 0.05.

### 4.7. Transcription Factor Activity Inference

Transcription factor (TF) activity was inferred from differential gene expression analysis comparing A-T patients and healthy controls using decoupleR (v2.17.0) [64] in combination with the CollecTRI regulon collection [65], accessed via the decoupleR package. CollecTRI comprises signed TF-target gene interactions for 1186 TFs. TF activity scores were estimated with the Univariate Linear Model (ulm) method, using DESeq2 “stat” values as input, which encodes the direction and significance of gene expression changes. Positive scores indicate higher inferred transcription factor activity in A-T samples relative to healthy controls, whereas negative scores indicate lower relative activity.

### 4.8. Clustering Analysis

For cluster analysis, we first used DESeq2 to perform a likelihood ratio test (LRT) to identify genes whose expression trajectories change significantly over time or are differentially expressed when considering healthy patients as a reference point. By comparing two statistical models, one that accounts for changes in gene expression between groups and one that assumes no group-dependent changes; the LRT evaluates whether including the group as a factor significantly improves the fit of the model. The groups in this case are three time-points for A-T patients and the healthy control group. A significant result suggests that the gene exhibits a dynamic expression pattern over time or is differentially expressed when considering healthy controls as a reference group.

We took the significant genes (adjusted *p*-value < 0.01) from the LRT results and clustered them using divisive hierarchical clustering implemented in the degPattern() function from DEGreport package [66]. The log_2_ normalized counts required by degPattern() were obtained using the *rlog* transformation from the DESeq2 library, with parameter blind = TRUE to avoid using information on the condition of the samples during variance estimation.

### 4.9. Over-Representation Analysis

Overrepresentation analysis was performed using the clusterProfiler package [67], with the following annotation databases: Gene Ontology Biological Process (GO:BP), Reactome (REAC) [68], Kyoto Encyclopedia of Genes and Genomes (KEGG) [69], and WikiPathways (WP) [70]. As the background gene set, we used all genes that remained after low count filtering and for which an Entrez ID was available based on the org.Hs.eg.db annotation package (v3.21.0) [71]. A false discovery rate (FDR) of 0.05 was used to filter significant results and all other parameters in the enrichment function from clusterProfiler package were set to the default. To homogenize results from all four inquired databases we computed a value Signal, which is defined as an equally weighted harmonic mean between the ratio of observed vs. expected genes in the set and −log(FDR). The intention of signal measure is to balance both metrics for more intuitive ordering of enriched terms.

### 4.10. Weighted Gene Co-Expression Network Analysis (WGCNA)

Raw counts from A-T samples at three time points were filtered by removing genes with low expression (counts < 15 in at least one third of the samples). Filtered counts were normalized using variant stabilization transformation (vst) from DESeq2. Clinical scores included A-T Neurological Examination Toolkit (A-T NEST), Scale for the Assessment and Rating of Ataxia (SARA), Gait Scale (GS) and neurofilament light levels (NfL), for further details on the tests and scores refer to Presterud et al. [19].

We constructed a signed weighted gene co-expression network using WGCNA package (v 1.74) in R [72]. The scale-free topology fit (R^2^) plateaued at β = 18 (R^2^ = 0.80), with mean connectivity = 84. In total, 34 distinct co-expression modules were identified, and module eigengenes (MEs) were correlated with clinical scores. *p*-values were adjusted for multiple testing across the set of observations and adjusted *p* < 0.05 was considered statistically significant. Within each module, we computed intramodular connectivity metrics: module membership (MM), defined as the correlation of the module eigengene and the gene expression profile and gene significance (GS), defined as the absolute correlation between a clinical score and the gene expression. Genes were considered highly connected and clinically relevant within a given module if they met the thresholds MM > 0.8 and GS > 0.5. The set of ISGs were identified/published by Kim and coworkers [73] and represents a set of ISGs that has been developed and validated for clinical diagnostic use.

## 5. Summary

Longitudinal transcriptomic analyses revealed that NR supplementation was associated with attenuation of interferon and innate immune gene signatures that were elevated at baseline. This reduction was evident within six months of treatment and persisted at later time points, suggesting a sustained effect on inflammatory signalling pathways. While the study was performed on a low number of patients and therefore does not allow causal inference, these observations are consistent with the hypothesis that restoration of NAD^+^ levels improves cellular stress responses and reduces chronic activation of DNA damage-induced inflammatory pathways. Given the proposed role of neuroinflammation in A-T pathogenesis, modulation of interferon signalling, perhaps in combination with addition to NR, may represent a relevant mechanism contributing to the observed clinical benefits.

## Data Availability

The raw data presented in this article are not readily available because of limitations due ethical standards on protecting person identifiable data. The de-identified processed counts files are available in Zenodo via the link https://doi.org/10.5281/zenodo.20662078.

## Supplementary Materials

The following supporting information can be downloaded at: https://www.mdpi.com/article/10.3390/ijms27135652/s1.
