# Supplementary material for: Transcriptional Profiling Shows Dampening of Interferon Gene Signatures by NAD+ Augmentation in Ataxia-Telangiectasia
Source: Int J Mol Sci. 2026 Jun 23;27(13):5652. doi: 10.3390/ijms27135652 (PMC13362275; doi:10.3390/ijms27135652)
Supplement: Supplementary file 1 [file ijms-27-05652-s001.zip › ijms-4350887-supplementary/supplementary figure.pdf]

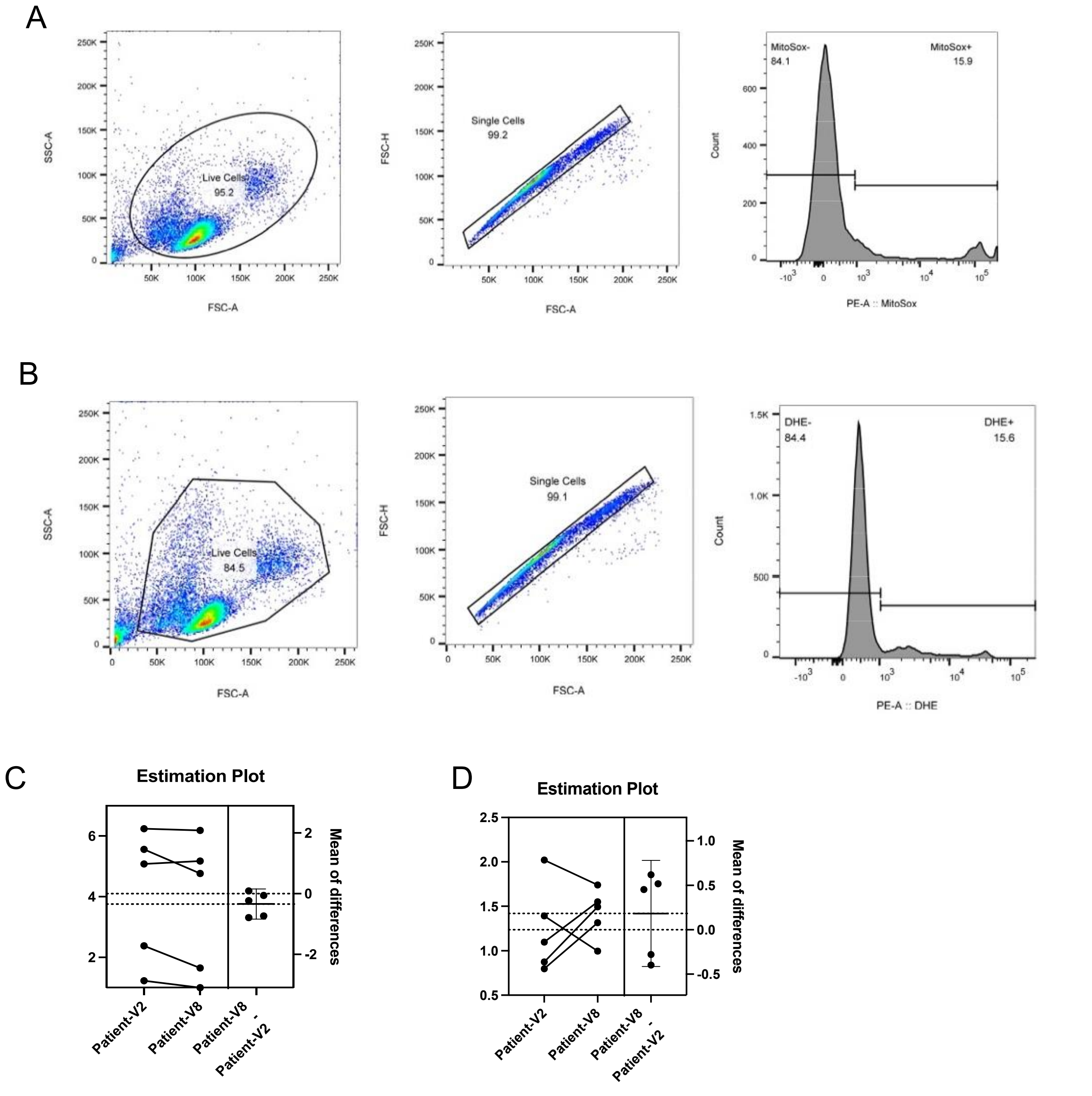

Supplementary Figure S1 Mitochondrial function in PMBC. A) Gating strategy cellular ROS. B) Gating strategy MitoSox. C) Effect size estimation cellular ROS (η^2 = 0.48) was large based on conventional thresholds (η^2 = 0.01 small, 0.06 medium, 0.14 large) D) Effect size estimation mitoROS (η^2 = 0.153).

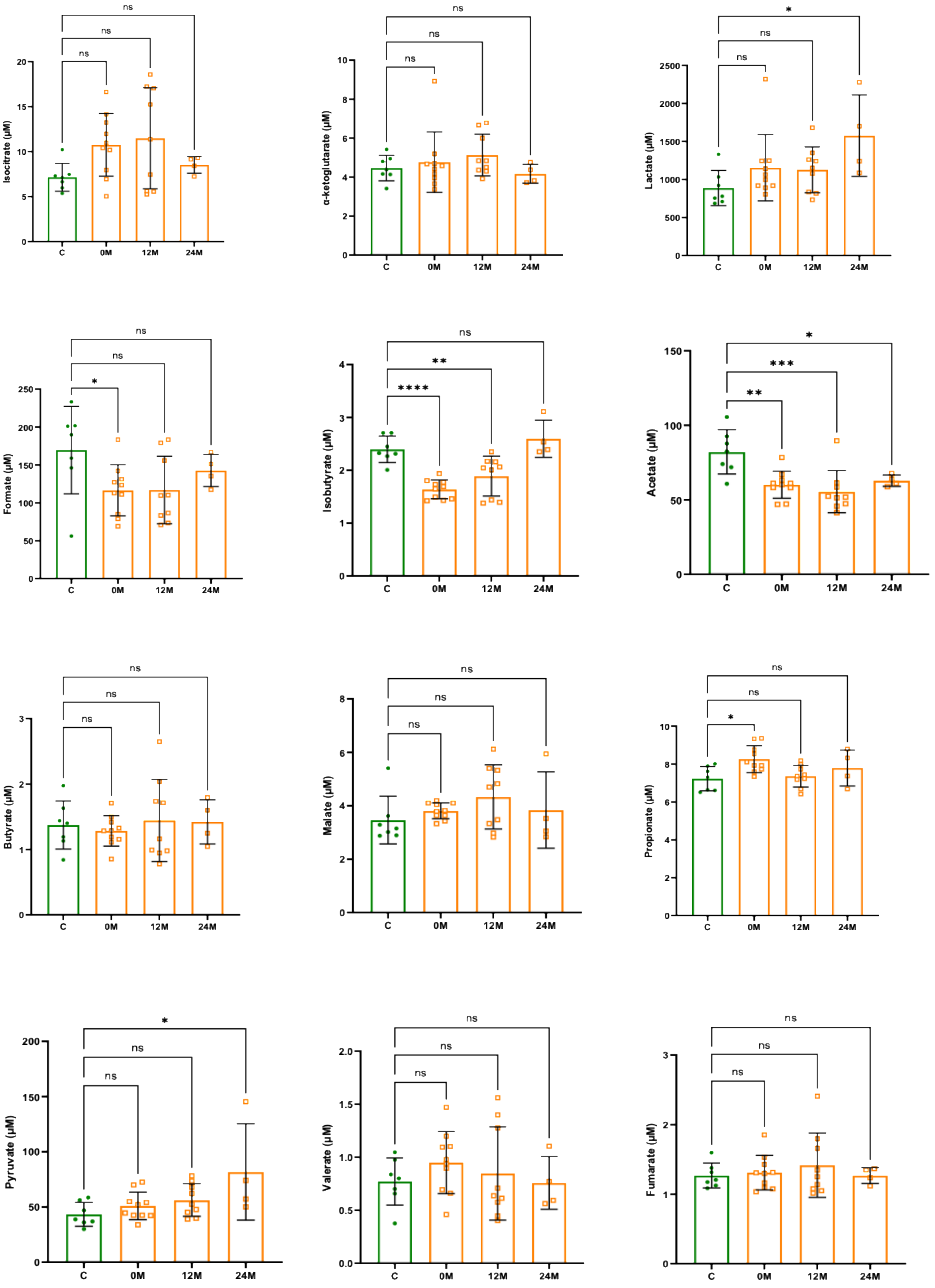

Supplementary Figure S2 Mitochondrial metabolites in plasma. Selected mitochondrial metabolie concentrations measured in plasma from healthy controls (C) and A-T patients at baseline (0M) as well as 12 (12M) and 24 (24M) months´ supplementation with NR

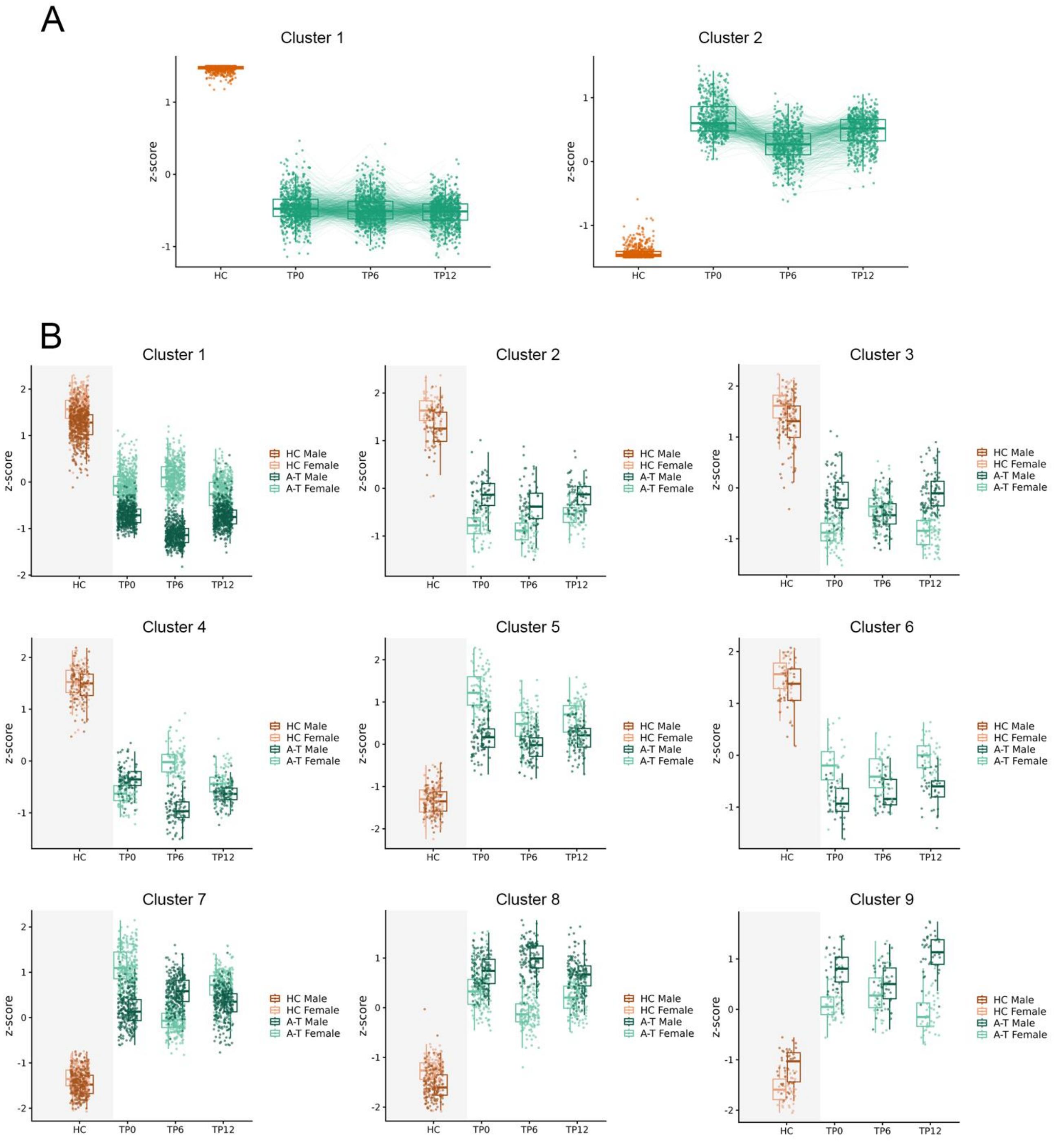

Supplementary Figure S3 Longitudinal gene expression dynamics in response to NR. Transcription profiling in healthy controls (C) and A-T patients at baseline (0M) as well as 12 (12M) and 24 (24M) months´ supplementation with NR. (A) Gene expression dynamics analysed from all study participants patients (A) and (B) all study participants separated by sex.

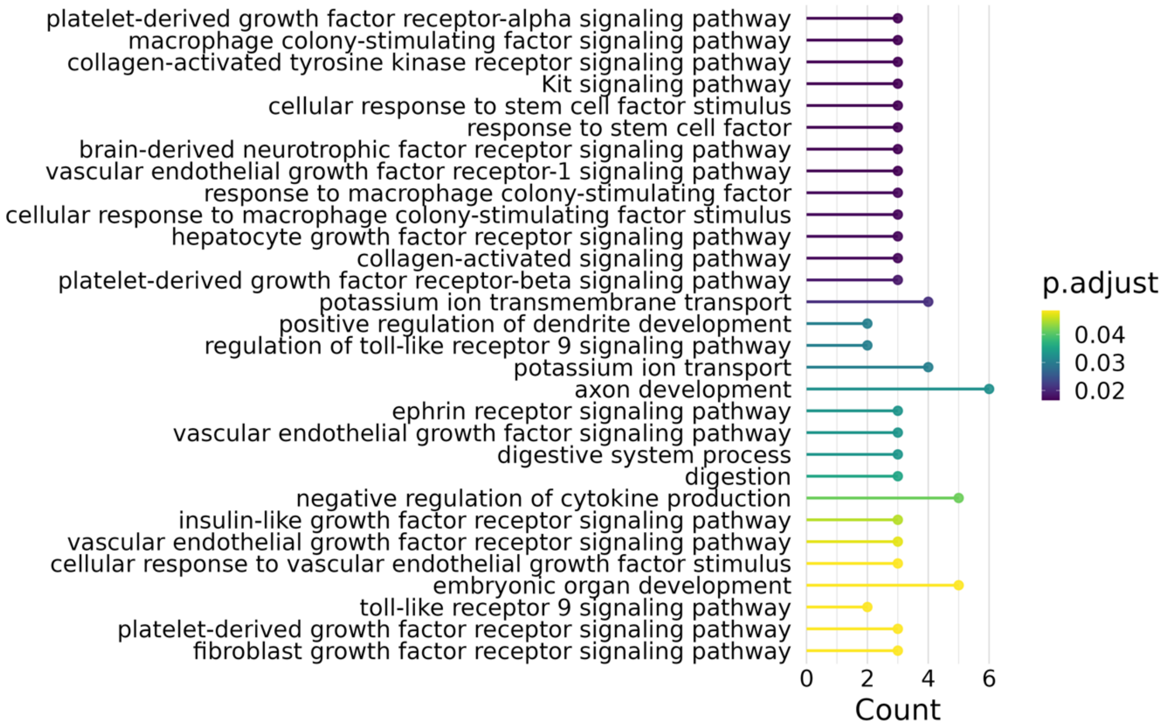

Supplementary Figure S4 Top pathways significantly enriched for module 18 (ME 18), identified using Gene Ontolgy Biological Process database.
